# Treatment of Pre-pubertal Patients with Growth Hormone Deficiency: Patterns in Growth Hormone Dosage and Insulin-like Growth Factor-I Z-scores

**DOI:** 10.4274/jcrpe.4125

**Published:** 2017-09-01

**Authors:** Megan Oberle, Adda Grimberg, Vaneeta Bamba

**Affiliations:** 1 The Children’s Hospital of Philadelphia, Division of Endocrinology and Diabetes, Philadelphia, PA, USA

**Keywords:** growth, insulin-like growth factor-I, Growth hormone deficiency, growth hormone therapy, clinical decision making

## Abstract

**Objective:**

To describe the range of insulin-like growth factor-I (IGF-I) z-score values (IGF-Iz) and growth hormone (GH) dose adjustments in pre-pubertal patients with GH deficiency (GHD) treated with GH in a single tertiary care center.

**Methods:**

This is a retrospective review of GH-treated patients of ages ≤9 years with GHD, seen in an endocrinology clinic in 2013-2014. Patient demographics and pre-treatment anthropometrics, GH treatment duration, IGF-Iz, and GH dosage (mg/kg/week) were extracted. Multipredictor linear regression was used to evaluate the associations between IGF-Iz and GH dosage and subject gender, race, insurance type, age, and clinical characteristics. Logistic regression was used to calculate the odds ratio of direction of GH dose adjustment (decrease/no change versus increase) and IGF-Iz category based on patient clinical characteristics, accounting for provider random effect.

**Results:**

Forty-one percent (57/139) of IGF-Iz were outside the “normal” range of between -2 and +2 standard deviation; the majority of IGF-Iz beyond the “normal” range (93%) were supraphysiologic [>+2 standard deviation score (SDS)]. Of the IGF-Iz >+2, 10/53 (18%) were followed by a GH dose increase and 30/53 (57%) had no dose change. Patient clinical characteristics and demographics did not significantly increase the odds of being in the IGF-Iz >+2 SDS category or having a dose increase in multipredictor logistic regression models.

**Conclusion:**

GH dosages and IGF-Iz varied, without significant patient clinical predictors. IGF-Iz was frequently supraphysiologic, and these levels often did not prompt a reduction in GH dose, likely influenced by a variety of factors. Our study emphasizes the need for better understanding of long-term safety and efficacy of maintaining supraphysiologic levels of IGF-Iz.

**What is already known on this topic?**

Insulin-like growth factor-I (IGF-I) can be used to monitor growth hormone (GH) therapy. Individualized IGF-I based dosing may be a more physiologic and objective approach to weight-based dosing. Guidelines by the Pediatric Endocrine Society recommend titrating GH to maintain IGF-I concentrations in the normal range for age and sex.

**What this study adds?**

Lack of evidence regarding dosing based on IGF-I z-score values (IGF-1z) contributes to variable clinical practice in GH dosing. This study examined GH prescribing practices and found a prevalence of supraphysiologic IGF-Iz. Our findings demonstrate the need to better understand not just factors that influence IGF-Iz but also the long-term effects of supraphysiologic IGF-Iz.

## INTRODUCTION

Historically, growth hormone (GH) therapy for pediatric patients with GH deficiency (GHD) has been guided by multiple clinical factors, including weight or body surface area, growth velocity, progression of skeletal maturation, side effects, and measurement of serum concentration of insulin-like growth factor-I (IGF-I). Individualized IGF-I-based GH dosing has been suggested as a more physiologic and objective approach to GH dose titration (1). Indeed, the Pediatric Endocrine Society guidelines recommend titrating GH dose “to maintain serum IGF-I concentration in the normal range for age and sex” (2). Reference ranges of IGF-I differ across commercial laboratories, but the recent advent of z-score reporting allows more standardized comparison of IGF-I levels across age, gender, pubertal status, and measuring laboratory. By comparing z-scores, clinicians compare normalized data to one another, just as one might compare body mass index (BMI) z-scores in children at different ages.

Despite these advances, the optimal target IGF-I level has not been established to balance height outcomes, safety, and cost. Short-term studies of IGF-I-based GH dosing have shown increased height outcomes when targeting an IGF-I z-score value (IGF-Iz) of 0 or +2 standard deviation score (SDS) compared to weight-based dosing (3,4). Although a greater increase in height was found to be associated with targeting an IGF-Iz of +2 SDS, higher doses of GH were required leading to supraphysiologic (>+2 SDS) IGF-I levels compared to targeting IGF-Iz of 0 (4). While there have been reports of adverse side effects, such as intracranial hypertension associated with higher doses of GH and supraphysiologic IGF-Iz, there is a lack of clinical data demonstrating a direct dose-response effect (5,6,7,8,9).

In practice, dosing decisions are influenced by subjective factors and therefore vary across clinicians and patients. Thus, we sought to retrospectively describe the range in IGF-Iz and patterns of GH dose adjustments in pre-pubertal patients with GHD treated with GH in a single tertiary care center and secondarily, to determine if GH dosage and IGF-Iz are associated with patient demographic and clinical factors.

## METHODS

The Children’s Hospital of Philadelphia Institutional Review Board approved this retrospective chart review with waiver of consent prior to data collection.

**Subjects:** The electronic health record (EHR) system was queried to identify all patients under age 9 years (chosen as a surrogate marker for pre-pubertal status) with ICD-9 code 253.3/ ICD-10 code E23.0 (pituitary dwarfism) or 253.2/E.23.6 (panhypopituitarism) who were treated with GH in the outpatient clinic of the Diagnostic and Research Growth Center of the Children’s Hospital of Philadelphia between January 1, 2013 and December 31, 2014. A member of the study team (M.O.) reviewed the records for each patient identified by the EHR query to confirm study eligibility. Patients were included if they had GHD defined by peak GH level <10 ng/mL on both arginine, clonidine, and glucagon stimulation testing or multiple pituitary hormone deficiencies (MPHD) with low GH or IGF-I concentrations based on age, sex, and reference range documented GH treatment during the study period, and measurements of IGF-I and IGF-Iz during the study period. IGF-Iz were reported by two commercial laboratories, based on their reference data, together with the absolute values of the IGF-I measurements. Patients were excluded if IGF-Iz were not reported. Patients also were excluded if they were Tanner stage 2 or greater on physical examination (10), were receiving active treatment for precocious puberty, or were being treated with GH for indications other than GHD.

**Procedures:** The following data were collected from the EHR: gender, age at the start of GH treatment, race/ethnicity, endocrinologist, insurance type, mid-parental height, baseline IGF-Iz, IGF-Iz on treatment, initial GH dose, GH dose at time of IGF-Iz measurement, and both pre-treatment and on-treatment weight, height (Ht), and BMI z-scores (z). Subjects’ gender-adjusted mid-parental heights were calculated and transformed into z-scores (mid-parental Htz) (10). Race/ethnicity by parental report was recorded at the time of the clinical visit. The commercial laboratory analyzing each IGF-Iz was also recorded. For each IGF-Iz obtained during the study period, the corresponding clinical notes were reviewed to determine if a GH dose adjustment was made. The majority of patients had IGF-I concentrations evaluated 2-4 times a year, so we also performed subgroup analyses on only the last IGF-Iz that was measured during the specified timeframe (last IGF-Iz).

### Statistical Analysis

Sample size was not calculated as subjects were drawn from a convenience sample of all cases that matched inclusion criteria. Statistical analyses were performed on two separate datasets: all IGF-Iz scores collected and the last IGF-Iz during the study period. Demographic and clinical characteristics were summarized by standard descriptive statistics. Continuous variables are presented as mean ± standard deviation (SD). The continuous variable, IGF-Iz, was categorized into three groups: low (IGF-Iz <-2 SDS), normal (IGF-Iz between -2 SDS and +2 SDS), and supraphysiologic (IGF-Iz >+2 SDS). Each IGF-Iz was also assigned to a category based on clinical decision: GH dosage increase, decrease, or no dose adjustment. Student’s t-test was used to compare IGF-Iz and GH dose by categorical variables (gender, race, and insurance type). Chi-squared test or Fisher’s exact test was used to compare categorical variables, including IGF-Iz categories and GH dose adjustment groups. Multipredictor linear regression, accounting for provider random effect, was used with the outcome variables IGF-Iz and GH dosage, and the potential predictors: gender, race, insurance type, age, and clinical characteristics. Logistic regression was used to calculate the odds ratio of direction of GH dose adjustment (decrease/no change versus increase) and IGF-Iz category based on patient clinical characteristics, accounting for provider random effect. All statistical calculations were performed on Stata Data Analysis and Statistical Software 14.0 (StataCorp LP, College Station, TX, U.S.). Statistical significance was defined as p-value ≤0.05.

## RESULTS

**Figure 1:**
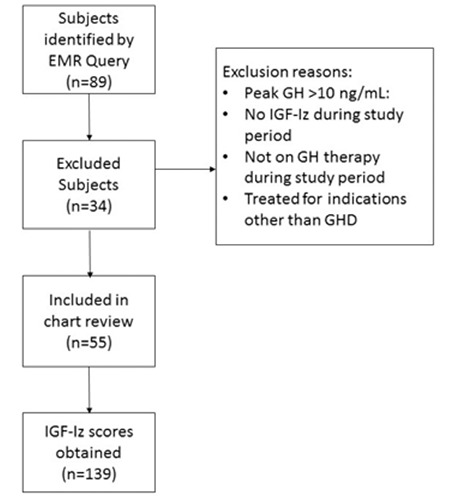
Flow diagram of included subjects and insulin-like growth factor-I z-score GH: growth hormone, GHD: growth hormone deficiency, IGF-Iz: insulin-like growth factor-I z-score

A total of 139 IGF-Iz were recorded from 55 subjects who met inclusion criteria (Figure 1). At the time of the last IGF-Iz assessment during the study period, subjects had a mean age of 6.1±1.5 years, and mean duration of GH therapy of 2.9±2.1 years. Sixty-four percent of subjects were male, 67% were white, and 65% had private insurance as their primary coverage (Table 1). Of the 55 subjects, 65% (36) had isolated GHD and 34% (19) had panhypopituitarism. Eighty-two percent (45) underwent stimulation testing and had peak GH levels less than 10 ng/mL. The 18% who did not undergo stimulation testing had MPHD (hypothyroidism, adrenal insufficiency, and/or diabetes insipidus) and low IGF-I and IGF-binding protein 3 concentrations based on age, sex, and reference range. Subjects with MPHD received replacement therapy for their other pituitary deficiencies per clinical routine. Clinical characteristics, including peak GH concentration on stimulation testing, baseline Htz, mid-parental Htz, or initial GH dosage, did not differ significantly between male and female patients.

### Patterns in Insulin-like Growth Factor-I Z-score and Growth Hormone Dosing

The mean of all IGF-Iz obtained during the study period was 1.57±1.8. The mean IGF-Iz was higher in males than females; this difference approached, but did not reach, statistical significance (1.79±1.9 vs. 1.20±1.3, p=0.06). The mean GH dose (mg/kg/week) prescribed during the study period was 0.28±0.9 and did not differ between males and females. The mean last IGF-Iz was 1.18±1.6, and the mean GH dose at last IGF-Iz assessment was 0.27±0.1 mg/kg/week. These measurements did not differ significantly between genders.

All 3 IGF-Iz below -2 SD were associated with subjects who had septo-optic dysplasia with central hypothyroidism, and 2 had low thyroxine at the time of their low IGF-Iz. After adequate thyroid replacement and normalization of thyroxine level, one subject’s IGF-Iz continued to be below -2 SD; the provider of this subject also documented non-adherence with GH at the time of the low IGF-Iz. The other subject did not have a repeat IGF-Iz obtained during the study period.

### Supraphysiologic Insulin-like Growth Factor-I Z-score

Of all 139 IGF-Iz measurements, 57 (41%) were outside of the generally accepted normal range and most of these (53/57, 93%) were supraphysiologic. More males were outside of the generally accepted normal range than females (47% vs. 30%, p=0.06).

### Predictors of Insulin-like Growth Factor-I Z-score

Using multipredictor linear regression accounting for provider random effect, an increase by 0.27 SDS in IGF-Iz was significantly associated with an increase by 1 SDS in most recent Htz adjusting for patient gender, race, insurance type, and age [p=0.03, 95% confidence interval (CI) 0.3, 0.5]. This association remained significant when including peak GH level on stimulation testing, pre-treatment Htz, GH dose, and mid-parental Htz in the linear regression model (ß=0.6, p=0.01, 95% CI 0.2, 1.0). Peak GH level on stimulation testing, GH dose, pre-treatment Htz, and mid-parental Htz were not significantly associated with IGF-Iz when adjusting for patient age, gender, race, and insurance type. Using logistic regression and controlling for provider random effect, patient clinical characteristics and demographics did not significantly increase odds of being in the supraphysiologic category. Logistic regression did not determine that etiology (isolated GHD vs. panhypopituitarism) was a significant predictor of IGF-Iz or GH dosage.

In the multipredictor models using IGF-Iz as the outcome, 49% of variation (R^2^) in the model came from patient demographics and clinical characteristics: gender, age at the start of GH treatment, race/ethnicity, provider, insurance type, mid-parental Htz, baseline IGF-Iz, IGF-Iz on treatment, baseline GH dose, GH dose at time of IGF-Iz measurement, pre-treatment weight, height, and BMIz, and on-treatment weight, height, and BMIz. Twenty-seven percent of variation was related to individual clinician. A remaining 24% of the variation in IGF-Iz was unidentified.

### Growth Hormone Dosage Titration

**Figure 2:**
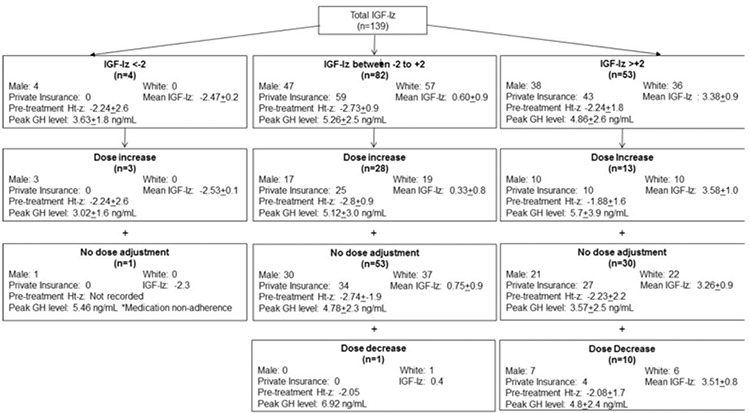
Flow diagram of all insulin-like growth factor-I z-score determinations arranged by insulin-like growth factor-I z-score category and dose adjustment category. Number of male subjects, white subjects, and subjects insured under private insured are listed for each category. Mean insulin-like growth factor-I z-score and Htz-scores are z-scores ±2 standard deviation. Peak growth hormone concentration is provided in ng/mL as mean value ± 2 standard deviation GH: growth hormone, IGF-Iz: insulin-like growth factor-I z-score

GH dose adjustments were categorized into three groups: no dose adjustment, dose increase, and dose decrease (Figure 2). For all IGF-Iz, the odds of a dose increase were not significantly associated with IGF-Iz category [p=0.8, odds ratio (OR) 1.1 95% CI 0.6, 2.0]. Of 82 measures of normal IGF-Iz, there was one instance (1/82, 1%) of subsequent dose decrease, 28 instances (28/82, 34%) of dose increase, and 53 instances (53/82, 65%) that were not associated with a dose change. Males were not more likely to receive a dose increase than females when IGF-Iz was normal (p=0.9, OR 1.0, 95% CI 0.4, 2.7).

When IGF-Iz were in the supraphysiologic category, 10/53 (19%) instances resulted in a dose decrease, 13/53 (25%) had dose increase, and 30/56 (56%) had no dose change. Patient clinical characteristics and demographics were not significantly associated with the odds of dose increase in the supraphysiologic IGF-Iz category. The odds ratio of an increase in GH dosage was not higher in males than females (p=0.95, OR 0.96, 95% CI 0.26, 3.53) when adjusting for patient demographics and provider.

Notably, one subject with low IGF-Iz was non-adherent to treatment (Figure 2). This was the only instance of the 4 with low IGF-Iz that did not result in a dose increase.

### Predictors of Growth Hormone Dosage

The mean GH dosage (mg/kg/week) did not differ significantly between IGF-Iz categories. Using multipredictor linear regression accounting for provider random effect, mid-parental Htz was found to be significantly associated with GH dosage when adjusting for patient gender, race, insurance type, and age (ß=-0.024, p=0.04, 95% CI -0.05, -0.001). Even when adjusting for other clinical characteristics (peak GH value on stimulation testing, pre-treatment Htz, IGF-Iz, and most recent Htz), an increase by 1 SDS in mid-parental Htz was associated with 0.036 mg/kg/week decrease in GH dosage (p=0.01, 95% CI -0.06, -0.01). Other clinical characteristics in the model (peak GH value on stimulation testing, pre-treatment Htz, IGF-Iz, and most recent Htz) were not found to be statistically significant (p>0.05). Patient demographics and clinical characteristics accounted for 47% (R^2^) of the variation in the prediction of GH dosage when accounting for provider random effect.

## DISCUSSION

Although the Pediatric Endocrine Society recommends titrating GH dose “to maintain serum IGF-I concentration in the normal range for age and sex” (2), we found that 41% of IGF-Iz obtained during a 2-year period at a large pediatric endocrinology center were outside of the laboratory-specified normal range of between -2 and +2 SDS, with the majority above +2 SDS (supraphysiologic). In addition, GH dose was increased in 25% (13/53) of instances where IGF-Iz was elevated, compared to 34% (28/82) when IGF-Iz was in the normal range. This observation suggests that IGF-z need not be the primary determinant of GH dose adjustments.

Htz and height velocity are clinical characteristics used to assess response to current GH dosage (2). Other clinical factors, such as mid-parental height, age, and gender may also influence clinical decision making. Mid-parental Htz was the only patient demographic and clinical characteristic tested in our study that significantly predicted GH dosage when adjusting for IGF-Iz. As mid-parental Htz increased, GH dosage decreased, suggesting that our patients with taller parents were more sensitive to lower doses of GH. This finding is consistent with other studies demonstrating that GH-deficient children can achieve comparable increases in growth velocity with smaller doses of GH than children with idiopathic short stature (4,5,11). Short children of tall parents are more likely to have more severe GHD, whereas short children of short parents might have familial short stature and are not truly GH deficient.

Clinician characteristics, such as age and gender, may also influence GH dose adjustment. Our study accounted for the bias of individual clinicians by accounting for healthcare provider in our statistical models. In a short informal survey of endocrinologists in our clinical practice, we found that the majority of providers base the initial GH dose on weight and then subsequently adjust the GH dose using the IGF-Iz in combination with other clinical factors (growth velocity, age, and pubertal status) to titrate GH therapy.

Although previous studies have suggested that physician beliefs and practices as well as consumer preferences play major, yet subjective, roles in referrals to subspecialists for short stature evaluation and even potential access to GH therapy (12,13,14,15), our findings do not demonstrate influence of patient gender on GH dose at the subspecialist level. However, there were more males than females with supraphysiologic IGF-Iz, with the result approaching statistical significance. A larger sample size may show gender bias in medical decision-making and GH dose titration.

**Figure 3:**
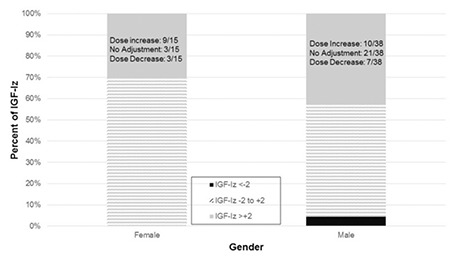
Proportions of insulin-like growth factor-I z-score in and outside of target range by patient gender. By Fisher’s exact test, insulin-like growth factor-I z-score obtained from male patients had a higher percentage of being outside of -2 to +2 standard deviation score (p=0.06). For those with supraphysiologic insulin-like growth factor-I z-score, decisions about growth hormone dose titration are provided GH: growth hormone, IGF-Iz: insulin-like growth factor-I z-score

The clinical variables included in our analysis, such as mid-parental height, Htz, and GH stimulation test results, were insufficient in the prediction of IGF-Iz associated with GH dosage. In our IGF-Iz prediction models, about half the variation (R^2^) was explained by patient demographics and clinical characteristics. A quarter of the variation was explained by individual clinician decisions (Figure 3), highlighting the degree of variability in GH titration amongst clinicians. The percentage of variation unexplained by the predictions models could be attributable to GH therapy adherence, height velocity, genetics, and co-morbidities or concomitant medications. Our statistical models also did not determine significant predictors of GH dosage or dose adjustment.

Similarly, prior studies have investigated the use of IGF-Iz and GH response prediction models in patients with GHD, such as the Pfizer International Growth Study (KIGS), the Gothenburg, and the Cologne models (16). Between 50-80% of the variation of growth velocity in the first year of GH treatment was explained by predictors such as age, gender, etiology of short stature, height velocity, change in height SDS, peak GH value on stimulation testing, serum IGF-I and IGF-binding protein 3 levels, and biomarkers of bone metabolism (16). IGF-I-based dose titration reduced this variation (16). Other factors contributing to the variation in IGF-Iz may include unidentified underlying conditions such as celiac disease, hypothyroidism, or nutritional deficits (16).

GH dose titration based primarily on IGF-Iz may prevent some subjectivity in GH dose adjustments, and because the target is generally normal IGF-I levels, decrease exposure to potential adverse side effects. Prescription of higher doses of GH and tolerance of supraphysiologic IGF-Iz may be done in an attempt to maximize adult height. However, it should be noted that short-term increases in height velocity may not translate to increase in adult height. Supraphysiologic IGF-Iz may accelerate bone age progression and with the subsequent loss of time for growth, result in a shorter adult height; a study using IGF-I therapy demonstrated that high doses of IGF-I may accelerate bone age (17).

At this time, there is limited clinical evidence to determine if long-term exposure to supraphysiologic IGF-Iz increases the risk of adverse events (9). GH therapy is associated with the development of increased insulin resistance, intracranial hypertension, slipped capital femoral epiphysis, and subsequent second neoplasms in patients with prior cancer treatment particularly radiation (5,6,7,18). The French subgroup of the Safety and Appropriateness of Growth hormone treatments in Europe (SAGhE) study found that higher doses of GH (greater than 50 µg/kg/day) were associated with increased all-cause mortality than expected in adults who had been treated with GH in childhood for isolated GHD, small for gestational age, or idiopathic short stature (7). These findings were controversial and not reproducible with other populations (8), which further highlights the need for additional research on the predictors and consequences of supraphysiologic IGF-Iz. Further research is necessary to balance positive outcomes of treatment with health care costs and adverse effects.

The retrospective nature of our study introduced several limitations. Most notably, this study was performed blinded to growth velocity, an important factor in GH treatment. Growth velocity is often used by clinicians in titrating GH dose. In our study, IGF-Iz and height measurements were often asynchronous in the EHR, and height measurements were taken by multiple specialties participating within a single patient’s care, leading to discrepant growth velocity calculations; therefore, we determined that growth velocity could not be accurately calculated. Despite this limitation, this study contributes information describing the range of IGF-Iz in the clinical setting. We were also limited in that adherence was not consistently ascertained and may influence variable bias, though likely less contributory to the supraphysiologic group. Selection bias may have been present since we only included data from commercial laboratories that reported IGF-Iz, and insurance preferences dictate the use of designated commercial labs, which may change over time. Our strengths include the use of IGF-Iz, which allows standardized comparisons of data across labs, inclusion of a diverse population, and the use of a prepubertal population, thereby eliminating effects of estrogen on the GH/IGF-I axis.

This report is a novel examination of GH prescribing practices by physicians and sheds light on the prevalence of supraphysiologic IGF-Iz. We took advantage of commercial lab z-score calculations to better understand GH prescribing practices in a large academic center. We did not find gender-specific differences in IGF-Iz and GH dosage when controlling for both provider and patient characteristics, although we had more male subjects who had IGF-Iz outside of the physiologic range. Our results suggest that multiple factors contribute to medical decision making related to GH surveillance and dosing.

## Figures and Tables

**Table 1:**
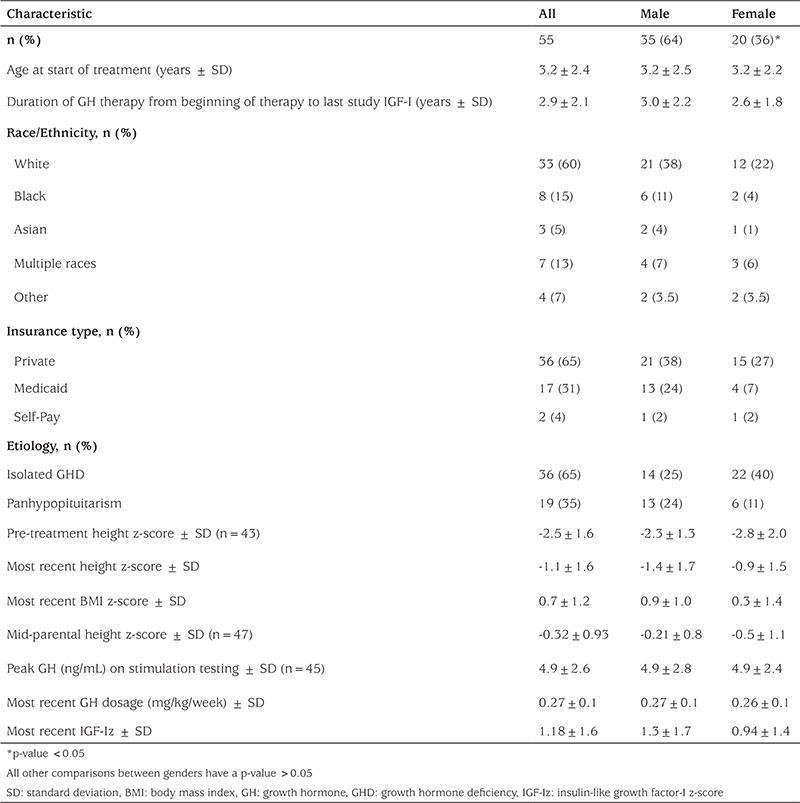
Clinical and demographic characteristics of subjects
